# Graphene Modified with Curcumin: A Novel Approach to Tailoring the Glass Transition of PVC

**DOI:** 10.1021/acs.jpcb.5c06706

**Published:** 2026-02-19

**Authors:** Sławomir Wilczewski, Aneta Woźniak-Braszak, Paweł Bilski, Jolanta Tomaszewska

**Affiliations:** † Bydgoszcz University of Science and Technology, Faculty of Chemical Technology and Engineering, Seminaryjna 3, 85-326 Bydgoszcz, Poland; ‡ Adam Mickiewicz University, Faculty of Physics and Astronomy, Uniwersytetu Poznańskiego 2, 61-614 Poznań, Poland

## Abstract

The glass transition temperature (*T*_g_) is a critical parameter that defines the thermal and mechanical functional limits of poly­(vinyl chloride) (PVC) and its nanocomposites. The influence of graphene (GN) and curcumin noncovalent-modified graphene (GN@CU), on the glass transition behavior and molecular dynamics of unplasticized PVC was investigated. A comprehensive set of thermal, mechanical, dielectric, and spectroscopic techniques, including differential scanning calorimetry (DSC), dynamic mechanical analysis (DMA), dielectric loss measurements, and solid-state ^1^H nuclear magnetic resonance (NMR) spectroscopy, was applied to investigate both local and segmental chain mobility in PVC-based nanocomposites. Spin–lattice relaxation times (*T*_1_), obtained from NMR measurements, provided molecular-level insights into chain dynamics associated with the glass transition. The results demonstrate that both GN and GN@CU restrict the mobility of the PVC chain, resulting in an increased *T*_g_ and composite stiffness. Especially, curcumin modification occurring according to the π–π interaction mechanism enhances filler dispersion and polymer–filler interfacial interactions, thereby further amplifying these effects. This work highlights the importance of integrating thermal, mechanical, dielectric, and NMR techniques to elucidate polymer-nanofiller interactions at the molecular level, which is crucial for designing PVC-based nanocomposites with improved thermal stability and mechanical properties suitable for demanding industrial applications.

## Graphical abstract

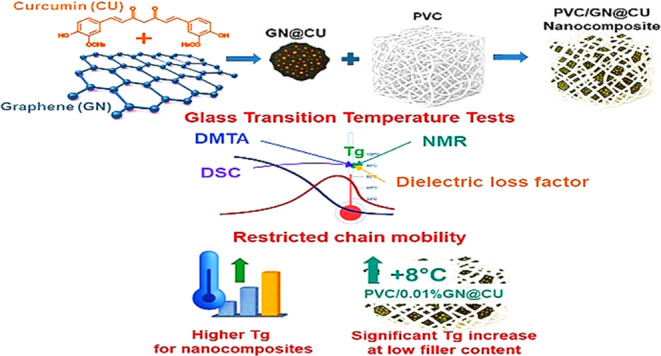

## 1 Introduction

The glass transition temperature (*T*_g_) is one of the most important factors limiting the temperature-dependent industrial applications of glass-forming polymers, polymer blends, and polymer composites. It is well-known that *T*_g_ is related to changes in chain mobility; therefore, below this temperature, all polymeric materials become rigid, exhibiting brittle fracture. On the other hand, above *T*_g_, polymers transition to a highly elastic solid state characterized by low modulus values, increased elongation, and enhanced impact resistance.^1–6^

The glass transition is accompanied by gradual changes in various polymer properties, including electrical and thermal conductivity, dielectric constant, specific volume, refractive index, thermal capacity, and others.^7,8^ The most commonly used techniques for determining the glass transition temperature are differential scanning calorimetry (DSC), thermomechanical analysis (TMA), dielectric analysis (DEA), and dynamic mechanical analysis (DMA).^2,3,9,10^

Nuclear magnetic resonance (NMR) spectroscopy is a valuable technique for studying the glass transition, as it allows the assessment of changes in molecular dynamics over a wide range of time scales.^11,12^ NMR enables the nondestructive investigation of segmental mobility, chain dynamics, and intermolecular interactions in polymer systems. The designated relaxation parameters, such as the spin–lattice *T*_1_ and the spin–spin *T*_2_ relaxation times, are sensitive to the local and segmental motion of polymer chains, providing valuable information on chain rigidity, confinement effects, and interactions between fillers and the polymer matrix.^13–19^ It should be noted that the value of the glass transition temperature for a given material can vary depending on the test method used and the measurement conditions, such as the measurement frequency, which is widely reported in the literature.^10,20–24^

Poly­(vinyl chloride) (PVC) belongs to the most frequently used polymers in the creation of nanocomposites, due to its beneficial properties, such as a comparatively low cost, extensively developed processing, wide possibilities of mechanical properties modification, and high environmental resistance. The restrictions of PVC applications are mostly related to the relatively high glass transition temperature of this polymer, resulting from strong polar interactions between chlorine and carbon atoms.^25,26^

Plasticizers are most commonly used to lower the *T*_g_ of PVC,^25–27^ however, other types of modifiers used in PVC compounds can also influence this temperature. Among them, carbonaceous nanofillers, such as graphene (GN), carbon nanotubes (CNTs), and fullerene C60, play a significant role in modifying PVC, also affecting its properties in the vitreous state.^21,24,28^ A direct impact on the molecular dynamics in PVC composites seems to be possible due to the nanosizes of these additives. The application of carbon nanofillers usually leads to an increase in *T*_g_ compared to the unfilled polymer, probably due to strong interaction between the nanoparticles and polymer chains, which restricts both segmental and long-range chain mobility. The shift of *T*_g_ toward higher temperatures may also indicate changes in the thermodynamic properties of the polymer. This effect is strongly influenced by various factors, including the dimensions of CNT and GN particles and their surface modifications. Moreover, the type and properties of PVC and the homogeneous distribution of carbonaceous nanofillers in the PVC matrix also play a significant role in determining the glass transition behavior.^24^

Previous studies have investigated the effect of multiwalled carbon nanotubes and multiwalled graphene on the *T*_g_ of PVC.^21,29^ An increase in *T*_g_ with increasing MWCNT content in the range from 0.01 wt % to 0.05 wt % was reported, with a saturation-like effect observed at the lowest MWCNT content used. These changes may indicate the influence of nanotubes on the mobility of the PVC chain. Furthermore, by increasing the measurement frequency from 1 Hz to 10^3^ Hz, a shift of *T*_g_ toward higher values was observed. By comparing the *T*_g_ values obtained from DMTA, dielectric, and DSC measurements, it was observed that dielectric measurements involving high-frequency periodic charging of the samples are the most sensitive method for detecting *T*_g_ changes in PVC/MWCNT composites.

Our conclusions are supported by scientific reports on the effect of carbon nanofillers on the glass transition of PVC, where *T*_g_ values depend not only on the properties of the polymer and the nanofiller introduced, but also on the preparation way of nanocomposites and the technique used to measure the glass transition temperature.^21^

We have also initiated research into the impact of multilayer graphene (MGN) on the thermal properties of PVC using nuclear magnetic resonance (NMR) spectroscopy. Initial studies on the glass transition were conducted using a pulse spectrometer operating at 25 MHz. The results indicate that the molecular dynamics of the PVC/MGN nanocomposite differ from those of pure PVC, with an increase in the glass transition temperature observed. This reduction in molecular mobility is assumed to be directly related to interfacial interactions between PVC and MGN, which restrict the segmental motion of the polymer chains.

Recent research trends involving graphene and graphene oxide are increasingly based on a green chemistry approach, which represents one of the most current and dynamically developing directions in the modification of graphene-based materials. This approach focuses on replacing conventional, often toxic chemical reagents with naturally derived compounds of low environmental impact. In recent years, polysaccharides, such as chitosan, have been widely investigated due to their ability to improve dispersion, hydrophilicity, and biocompatibility of graphene materials, particularly for biomedical applications.^30^ An important group of natural modifiers are plant-derived polyphenols, especially tannic acid, which can be used both for surface functionalization and more ecological reduction of graphene oxide.^31,32^

Growing attention has also been devoted to curcuminoids, which enable effective graphene modification through π–π interactions and the presence of multiple functional groups, improving compatibility with polymer.^33,34^ In parallel, methods for the “green” reduction of graphene oxide using plant extracts have been developed, where natural compounds act simultaneously as reducing and stabilizing agents.^35^ This green approach significantly reduces process toxicity and aligns with the principles of sustainable development for advanced nanomaterials.

In our previous studies, which align with current trends in green modification of graphene, we determined the effectiveness of graphene modification with curcumin (CU) and the impact of such a modified nanofiller on the structure as well as the physical and mechanical properties of PVC nanocomposites in the form of films.^34,36,37^ For a complete characterization of such materials, understanding the effect of curcumin-modified graphene on the glass transition of the polymer matrix macromolecules is still lacking. This knowledge is important in terms of the application of such PVC-based nanocomposites with graphene. In our previous studies, which align with current trends in green graphene modification, we determined the effectiveness of graphene modification with curcumin (CU) and the impact of such a modified nanofiller on the structure as well as the physical and mechanical properties of PVC nanocomposites in the form of films.^34,36,37^ For a complete characterization of such materials, understanding the effect of curcumin-modified graphene on the glass transition of the polymer matrix macromolecules is still lacking. This knowledge is important in terms of the application of such PVC-based nanocomposites with graphene.

Therefore, this study aims to evaluate the influence of carbonaceous nanofillers, such as graphene (GN) and curcumin-modified graphene (GN@CU), on the flexibility or rigidity of the unplasticized PVC chains. The determination of the glass transition temperature values was realized using dynamic mechanical thermal analysis, dielectric loss measurements, and differential scanning calorimetry. Additionally, solid-state ^1^H NMR measurements were employed to further investigate the molecular dynamics of PVC chains. The temperature dependence of the spin–lattice relaxation times (*T*_1_) provides insights into segmental chain mobility, which is directly related to the glass transition temperature. Therefore, measuring relaxation times enables phase transitions to be studied, particularly the transition from the rubbery to the glassy state.^14,16,38^

## 2 Methodology

### 2.1 Materials

Graphene from USA Graphene Laboratories Inc. (flake thickness 1.6 nm, flake length 10 μm, specific surface area between 400 and 800 m^2^ g^–1^); unmodified suspensive poly­(vinyl chloride) Neralite 601 from Spolana s.r.o. Anwil S.A. group. (Czech Republic) (*K* value 59–61, bulk density 0.56–0.63 g cm^–3^, specific density 1.39 g cm^–3^, and purity 97%); Curcumin from the rhizome of *Curcuma longa* L. (Sigma-Aldrich, St. Louis, MO, USA); Tetrahydrofuran (THF) (Chempur, Piekary Śląskie, Poland); Methanol (Chempur, Piekary Śląskie, Poland).

### 2.2 Methods

#### 2.2.1 Graphene Modification

Graphene was surface-modified using curcumin by preparing a solution of CU in THF with a concentration of 2.25 mg cm^–3^. In the next step, graphene was added to the solution to achieve a concentration of 1.5 mg cm^–3^. Graphene was ultrasonically dispersed for 2 h (frequency 20 kHz), 40% amplitude, temperature 23 °C using Bandelin’s SONOPULS rod-shaped homogenizer. Subsequently, the dispersion was mixed for 24 h using a magnetic stirrer at 250 rpm. After this time, the modified graphene was centrifuged and washed with methanol to remove undeposited curcumin. The operation was repeated four times. The obtained material was dried for 65 h at 45 °C to remove residual solvents. Finally, graphene surface-modified by curcumin (GN@CU) was obtained.

#### 2.2.2 Obtaining PVC/GN Nanocomposites

Poly­(vinyl chloride) nanocomposites with graphene were prepared by the solvent casting method. For this purpose, 3 wt % solutions of PVC in THF were prepared. Subsequently, graphene was dispersed in solution using ultrasound, according to the conditions presented in the “Section 2.2.1”; however, the dispersion time was 60 min. The resulting dispersions were poured onto Petri dishes, and the solvent was evaporated from them at 50 °C for 24 h. The obtained nanocomposite films were dried under vacuum (to remove residual solvent) at 50 °C for 2 weeks. Materials containing GN@CU as filler and unmodified matrix were obtained under the same conditions. Nanocomposites containing 0.01, 0.1, and 1 wt % filler in PVC matrix were produced. All samples were coded in the same way, for example, materials containing 1 wt % GN were designated as PVC/1%GN, while those containing 1 wt % of GN@CU were labeled as PVC/1%GN@CU.

#### 2.2.3 Characterization Methods of Fillers

Transmission electron microscopy (TEM) was used to evaluate the morphology of the fillers used, and a Zeiss Libra 120 Plus microscope (Stuttgart, Germany) operating at 120 kV was used for the study. Samples (GN, GN@CU) for the study were placed on a copper grid coated with a layer of Formvar polymer films. Scanning electron microscopy (SEM) was used as a second technique. A Zeiss Crossbeam 350 microscope was used. The SEM technique was also used to observe cryogenic breakthroughs of nanocomposites containing 1% fillers to evaluate the structure of the resulting materials. The samples for observation were sputter-coated with a layer of gold.

The following spectroscopic methods were also used to characterize the used fillers and asses the graphene modification: Raman spectroscopy, where spectra were collected using a SENTERRA II Raman microscope (Bruker Corporation, Billerica, MA, USA) at a laser wavelength of 785 nm. Samples were measured in 50 replicates of 50 s (20 in subsequent control measurements). The aperture was 50 μm, the objective was 50 mm, and the laser beam power was 0.1 mW. Each sample was measured at least 80 times at different locations, and the displayed spectrum represents the average of these measurements.UV–vis spectroscopy where absorption studies were carried out using a UV–vis/NIR spectrometer (PerkinElmer Lambda 1050+) in a quartz cuvette. Absorption spectra were recorded from 210 to 800 nm for GN and GN@CU dispersions and curcumin solution in methanol. The concentration of CU was 0.008 mg cm^–3^ while that of the filler dispersion was 0.03 mg cm^–3^. The experimental results were normalized in the range of 0–1 using OriginLab software. X-ray photoelectron spectroscopy (XPS): VG Scienta R3000 hemispherical analyzer was used to perform XPS measurements of GN and GN@CU samples. The experiments were conducted using monochromatic Al Kα radiation (*h*ν = 1486.6 eV), the X-ray source operating at 25 W, 15 kV, 100 μm spot, pass energy 23.5 eV, energy step 0.1 eV. The obtained spectra were analyzed with CasaXPS software, using a set of sensitivity coefficients specific to the equipment. The Shirley background correction and Gaussian–Lorentzian peak shapes were used to deconvolute all spectra.

The thermal stability of GN and GN@CU was evaluated by the thermogravimetric method. The tests were performed with a TG 209 F3 Tarsus apparatus (Netzsch). Samples were heated at a rate of 10 °C min^–1^ in an open ceramic crucible under a nitrogen atmosphere over a temperature range of 30 to 700 °C. Differences in the masses of the samples at 700 °C were used to determine the amount of curcumin deposited on the graphene surface.

#### 2.2.4 Determination of the Glass Transition

The determination of the glass transition temperature of the nanocomposites of PVC with GN was realized by means of dynamic mechanical thermal analysis (DMTA), dielectric loss measurements, and differential scanning calorimetry (DSC). The NMR technique was used to study the molecular dynamics. The DMTA measurements in a bending mode, operating at frequencies *f* = 0.1, 1, and 10 Hz, were performed by means of Netzsch DMA 242. The heating rate was 2 °C min^–1^ in the range from 20 to 120 °C, and the temperature at which tan δ reaches its maximum was taken as the glass transition temperature. The dielectric loss measurements (tan δ_dl_) were performed using the Schering E315A bridge at a field frequency of 1000 Hz. The heating rate was 2 °C min^–1^ in the temperature range between 20 and 110 °C, and the glass transition temperature was determined at the maximum of the dielectric loss tangent.

The glass transition temperatures for pure PVC, PVC/GN, and PVC/GN@CU nanocomposites were determined using differential scanning calorimetry with a properly calibrated PerkinElmer DSC 8000 calorimeter. Initially, the samples were cooled from ambient temperature to 0 °C and then heated to 120 °C at a rate of 10 °C min^–1^. Subsequently, the cooling/heating cycle was repeated. The glass transition temperatures were estimated as the midpoint of the step change in the thermogram.

The solid-state ^1^H NMR measurements of the spin–lattice relaxation times (*T*_1_) in the laboratory frame were performed using a pulse spectrometer operated at 25 MHz (El-Lab Tel-Atomic). The values of the spin–lattice relaxation times *T*_1_ were measured employing the saturation recovery sequence over the temperature range from 80 to 370 K. The recovery of magnetization Mz as a function of time t followed a single-exponential behavior and was described by the equation Mz­(*t*) = *M*_0_·(1 – exp­(−*t*/*T*_1_)), where *M*_0_ is the equilibrium magnetization. The accuracy of the measurements was maintained within 10%.

## 3 Results and Discussion

### 3.1 GN and GN@CU Morphology

TEM and SEM images confirmed that the graphene used in this study was a few-layer material (Figure 1A,A1). Due to its relatively large flake size and small thickness (i.e., number of layers), the graphene exhibited a wrinkled and crumpled morphology, which is typical for materials with such dimensional properties.^39–41^ The TEM image of modified graphene (GN@CU) (Figure 1B) revealed the presence of curcumin forming flower-like structures on the GN flakes. Additionally, SEM images (Figure 1B1) showed spherical white curcumin particles deposited on the graphene surface.^20,42^ These observations indicate that the applied modification method effectively facilitated the deposition of curcumin onto the graphene flakes.

**Figure 1:**
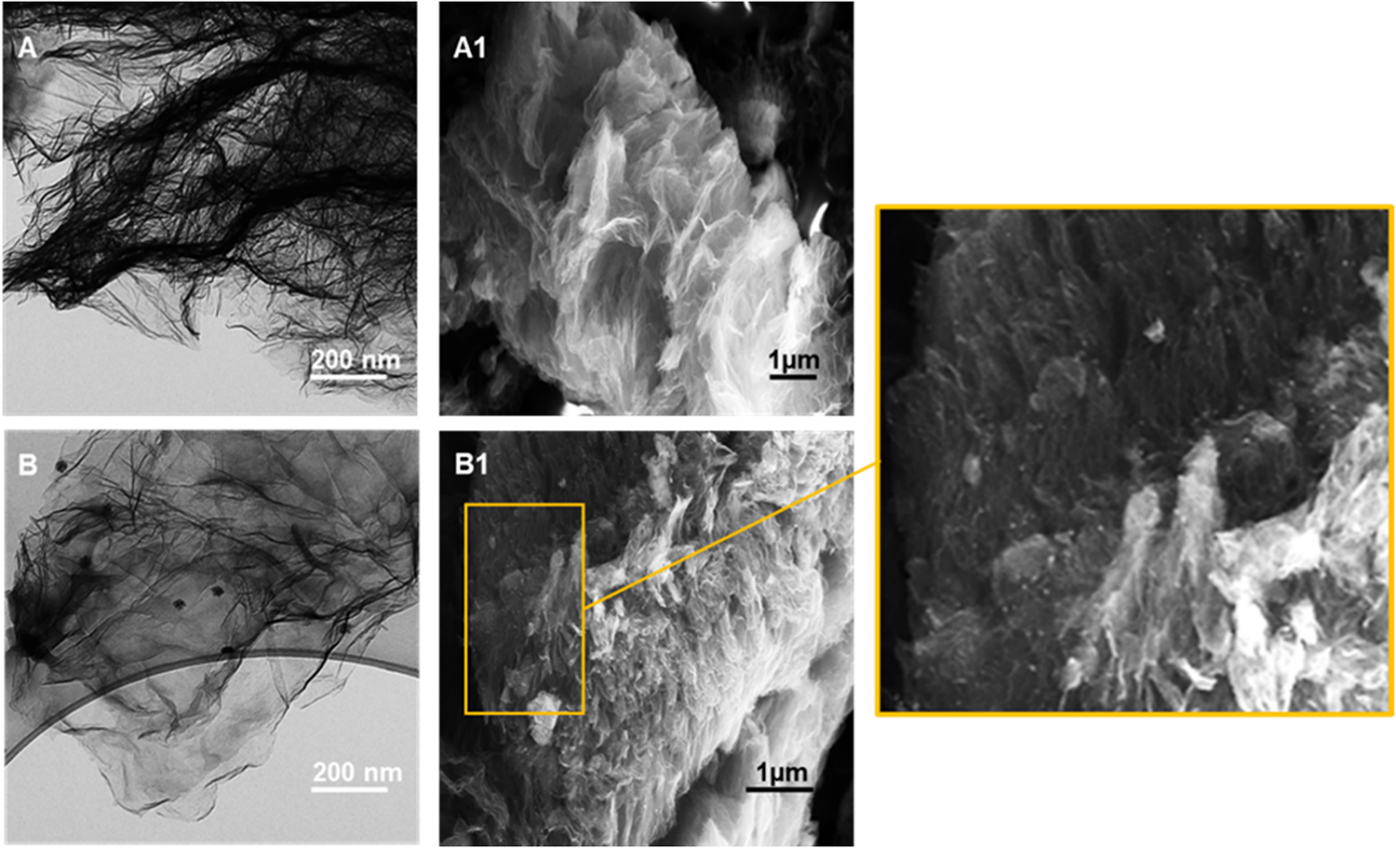
TEM images of graphene (A), graphene modified by curcumin (B), SEM images of graphene (A1), and graphene modified by curcumin (B1).

### 3.2 Fillers Spectroscopic and Thermal Properties

Raman spectroscopy is a valuable technique for studying the structure of carbon materials. In the case of graphene, two characteristic bands appear in its spectrum: the D band, associated with structural disorder, and the G band, corresponding to the crystalline structure of graphite. Additionally, Raman spectroscopy provides important information about the number of layers in the graphene material used.^43,44^ In the Raman spectra of GN and GN@CU (Figure 2A), both characteristic bands were observed. The D band appeared at 1309 cm^–1^ for graphene and 1310 cm^–1^ for curcumin-modified graphene, while the G band was detected at 1598 cm^–1^ and 1600 cm^–1^, respectively. The intensity ratio of the D and G bands (*I*_D_/*I*_G_) is commonly used to determine the size of graphene domains,^45^ with its value being inversely proportional to the crystallite size. For the materials presented here, the *I*_D_/*I*_G_ ratio was 1.8 for GN and 1.9 for GN@CU, indicating a high degree of structural defects, which increased further after modification. When graphene is used as a filler in polymer composites, a high degree of defectivity can be beneficial, as it enhances the penetration of the polymer matrix into the filler, leading to improved interactions at the filler–polymer interface.

**Figure 2:**
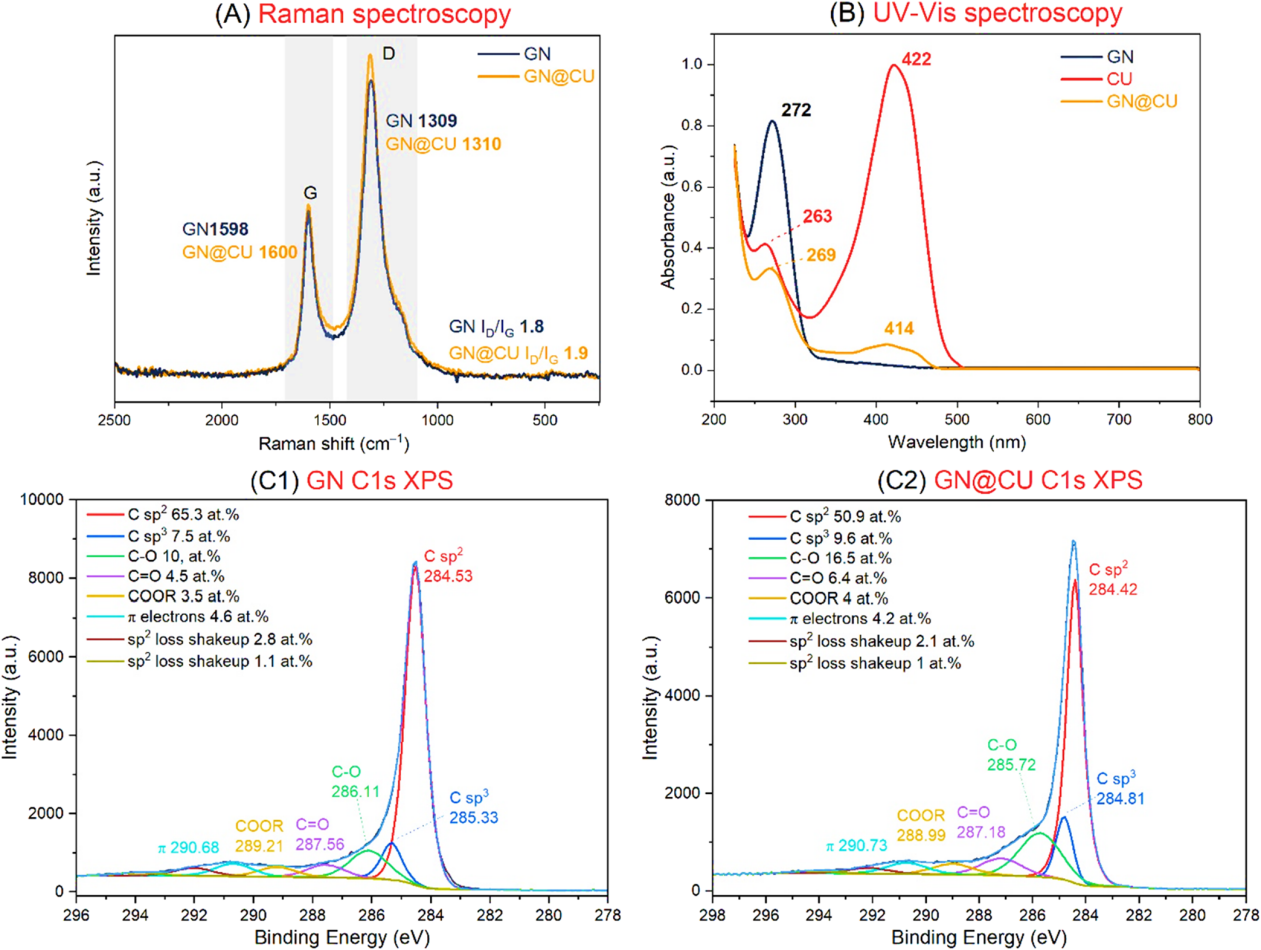
Spectroscopic properties of GN nanofillers: (A) Raman spectra of GN and GN@CU, (B) UV–vis spectra of GN, GN@CU and CU, (C1) XPS spectrum of GN, (C2) XPS spectrum of GN@CU.

The UV–vis spectroscopy studies presented in Figure 2B, recorded for a curcumin (CU) solution and dispersions of GN and GN@CU in methanol, revealed distinct absorption features. The absorption maximum for graphene was observed at 272 nm, which is attributed to π–π* transitions of C=C bonds.^46,47^ In the UV–vis spectrum of curcumin, two absorption bands were identified: one at 263 nm, associated with π–π* transitions of C=C bonds, and another at 422 nm, corresponding to the *n*–π* transition of C=O groups.^45,45^ In the GN@CU spectrum, absorption bands appeared at 269 and 414 nm, which were assigned to the π–π* transition of C=C bonds and the *n*–π* transition of C=O bonds, respectively. The presence of the 414 nm band, characteristic of curcumin, in the modified graphene spectrum confirms the effective functionalization of the graphene surface.^48^ Additionally, the position of the 269 nm band in GN@CU, falling between the maxima observed for GN (272 nm) and CU (263 nm), further supports the occurrence of π–π* interactions between graphene and curcumin.^48–50^

X-ray photoelectron spectroscopy (XPS) was employed to precisely characterize the functionalized graphene surface. The XPS spectra of GN and GN@CU are presented in Figure S1 (Supporting Information). The spectrum of modified graphene (GN@CU) exhibits a significant increase in the intensity of the O 1s band compared to GN, which is attributed to the presence of curcumin (CU) containing oxygen functional groups on the material’s surface.^51^

The deconvolution of the O 1s peaks and the XPS characteristics of GN and GN@CU sheets are shown in Figure S2 and Table S1 (Supporting Information). Given the use of graphene material, sp^2^ hybridization of carbon atoms was primarily expected. However, as shown in Figure 2C1, the inclusion of sp^3^ hybridization was necessary for peak fitting. The presence of sp^3^-hybridized carbon is attributed to defects on the GN surface, as confirmed by Raman spectroscopy.^52,53^

The observed C sp^2^ and C sp^3^ peak separation of 0.8 eV (predominantly in unmodified graphene) aligns with literature-reported values for thin carbon films.^53–59^ Additionally, the C sp^2^ peak, located around 284.5 eV, is characteristic of this type of material^55^ and falls within the typical literature range (284.25–285 eV).^59,60^ The positions of other carbon peaks (Figure 2C2 and Table S1) associated with carbon functional groups exhibit expected shifts relative to the main C sp^2^ peak.^61^ Given that curcumin contains carbon–oxygen functional groups, the XPS spectrum of GN@CU (Figure S1) confirms an increase in oxygen content (O 1s), from 0.6 atom % in GN to 5.3 atom % in GN@CU, further validating the successful deposition of curcumin on the graphene surface.

Moreover, changes in π-electron contributions (4.6 atom % for GN vs 4.2 atom % for GN@CU), as observed in the C 1s spectra (Figure 2C1,C2 and Table S1), indicate that the interaction between graphene and curcumin is primarily physical, driven by noncovalent π–π stacking modifications.^58,62^

Due to the thermal sensitivity of poly­(vinyl chloride) (PVC), fillers and their modifiers must exhibit high thermal and chemical stability. A low thermal stability of the filler can also influence the glass transition temperature of the polymer. Additionally, since PVC processing requires temperatures close to 200 °C,^63,64^ only modifiers stable at these temperatures can be effectively applied. The thermal stability of graphene in a nitrogen atmosphere enables the use of thermogravimetric analysis (TGA) to determine the amount of curcumin deposited on the graphene surface. The analysis was conducted over a temperature range of 30–700 °C. The results (Figure 3) demonstrated that graphene remained thermally stable throughout this range, while curcumin (CU) was stable up to 250 °C, with decomposition occurring between 250 and 550 °C. The initial degradation (up to ∼320 °C) was attributed to the breakdown of −OH and –OCH_3_ groups, while the decomposition of the remaining curcumin structures occurred at higher temperatures.^65–67^ GN@CU exhibited stability up to approximately 340 °C, with further decomposition linked to the degradation of the organic modifier. The high thermal stability of both GN@CU and CU, well above 200 °C, confirms their suitability for PVC modification. The curcumin content on the graphene surface was determined by comparing the reference mass loss of GN with the mass loss of GN@CU at 700 °C. Calculations indicated that the curcumin loading on graphene was 11.2 wt % (Figure 3).

**Figure 3:**
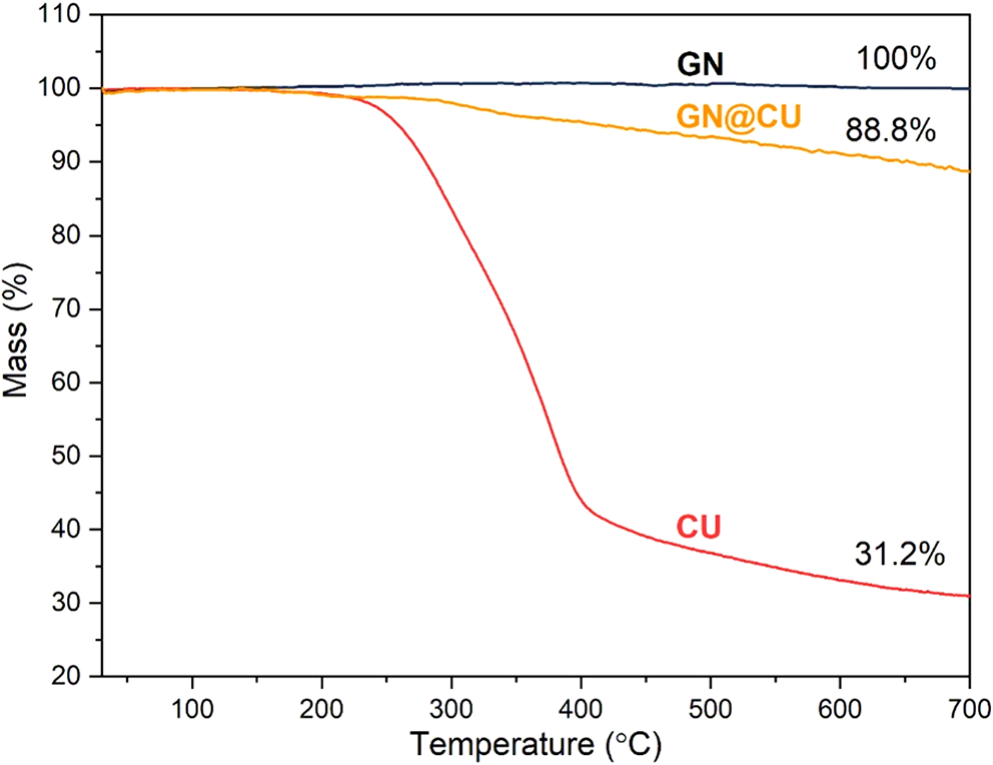
TGA of curcumin, GN, and GN@CU.

### 3.3 Nanocomposites Structure

Problems with the homogeneity of PVC nanocomposites with graphene are particularly evident in materials with a higher filler content (typically above 0.5%) in the matrix.^37,68^ Therefore, in this study, structural observations were conducted for materials containing 1 wt % GN. The addition of CU resulted in a significant enhancement of the homogeneity of PVC/GN nanocomposites, observable even at the macroscopic scale. Figure 4A–C show digital photographs of PVC, PVC/1%GN, and PVC/1%GN@CU, respectively. As observed, materials with modified graphene as a filler exhibit a homogeneous structure. To analyze the structure of the nanocomposites in greater detail, SEM observations were conducted (Figure 4A1–C1). The materials obtained did not exhibit any structural defects, such as air bubbles. The PVC fracture displayed characteristics typical of the brittle fracture of thermoplastics. The obtained nanocomposites exhibited a structure distinct from that of unmodified PVC.^53,68^ The PVC/1%GN nanocomposite consisted mainly of graphene agglomerates embedded in the polymer matrix. In contrast, the PVC/1%GN@CU nanocomposite had a homogeneous structure, as can be seen in Figure 4C1, where the individual graphene flakes were completely covered by PVC. This material’s structure appears more irregular, resembling rough sea waves. The absence of distinct boundaries between the matrix and the filler clearly indicates the homogeneity of these nanocomposites and a stronger interaction between the polymer matrix and the filler.^37,69,70^ This effect is attributed to the use of the modifier. The deposited curcumin remained on the graphene flakes, as evidenced by the spherical white shapes observed on them (close-up of Figure 4C1).^42^

**Figure 4:**
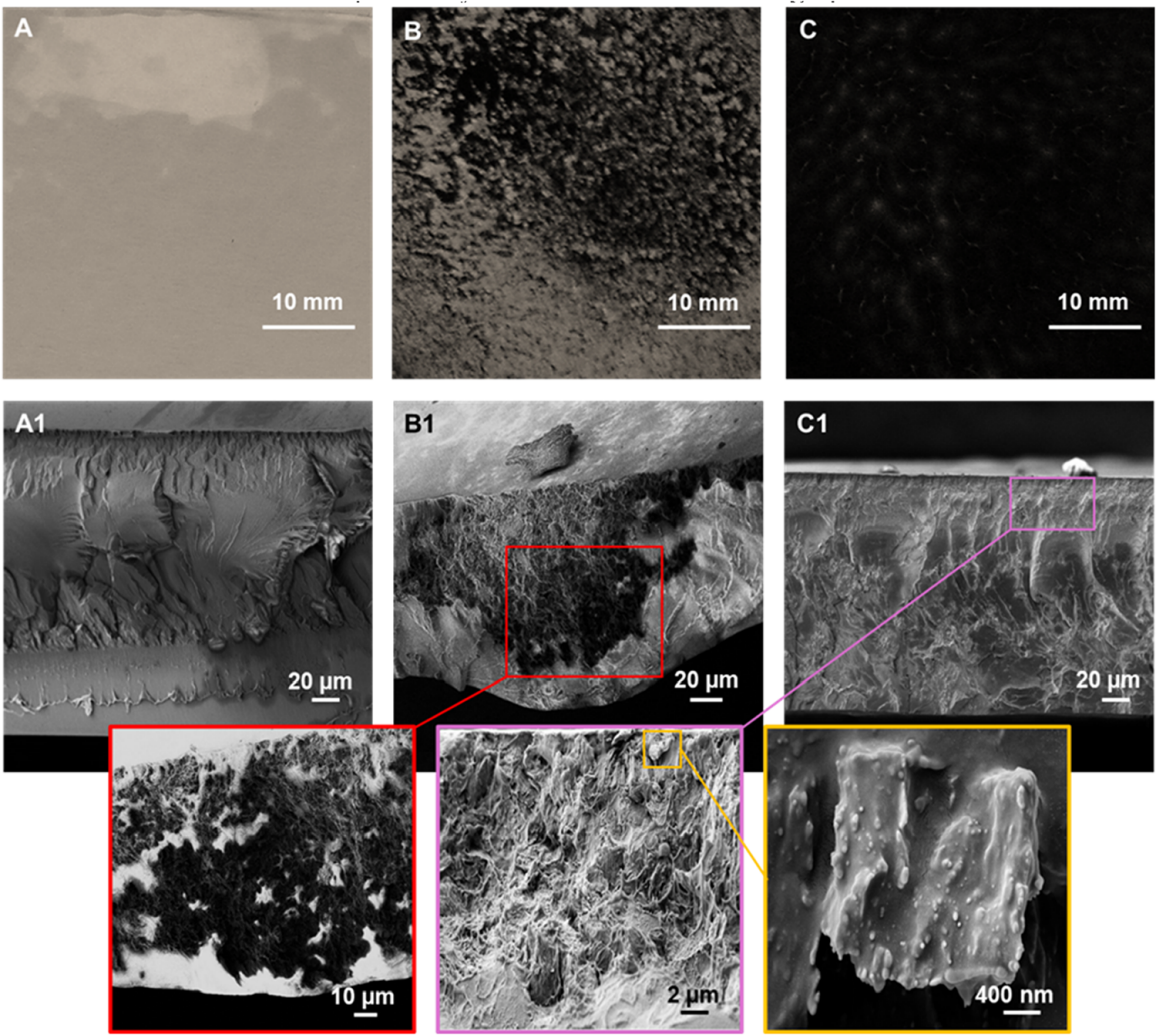
Structure of nanocomposites, ***digital photos:*** (A) PVC, (B) PVC/1%GN, (C) PVC/1%GN@CU, ***SEM images:*** (A1) PVC, (B1) PVC/1%GN, (C1) PVC/1%GN@CU.

For the purposes of the results presented in this work, only observations of the structure of the obtained nanocomposites are shown to demonstrate the increased uniformity of these materials after the application of the modifier. However, as indicated by our previous studies,^34,36,37^ the interactions between the filler and the matrix have a physical nature, even after the surface modification of GN, which we confirmed using FT-IR and Raman spectroscopy. The use of CU as a modifier also positively affects the exfoliation of graphene in the PVC matrix.

### 3.4 Glass Transition of Nanocomposites

Figure 5A shows the values of the glass transition temperature for neat PVC and PVC nanocomposites containing graphene and modified graphene as a function of frequency, which was 0.1, 1, and 10 Hz, respectively. For all materials, an increase in the glass transition temperature with increasing frequency was observed, which is characteristic behavior for polymeric materials. Over the entire analyzed frequency range, materials containing GN@CU exhibited higher average glass transition temperatures compared to unmodified PVC. In the case of materials containing GN, an increase in *T*_g_ was observed only for samples with 0.01 wt % filler content, whereas higher graphene loadings resulted in a decrease in the glass transition temperature at 0.1 Hz or no statistically significant change relative to poly­(vinyl chloride). The application of curcumin as a modifier had a beneficial effect on increasing the glass transition temperature also at higher filler loadings (0.1 and 1 wt %), as evidenced by higher average *T*_g_ values of these materials compared to both neat PVC and nanocomposites with the same content of unmodified GN. These materials also exhibited lower standard deviations, which can be attributed to their increased structural homogeneity. Nevertheless, regardless of frequency and filler type, the highest increase in *T*_g_ was observed for materials containing 0.01 wt % filler. Therefore, the linear trend model shown in Figure 5B was constructed based on materials containing 0.01 wt % filler.

**Figure 5:**
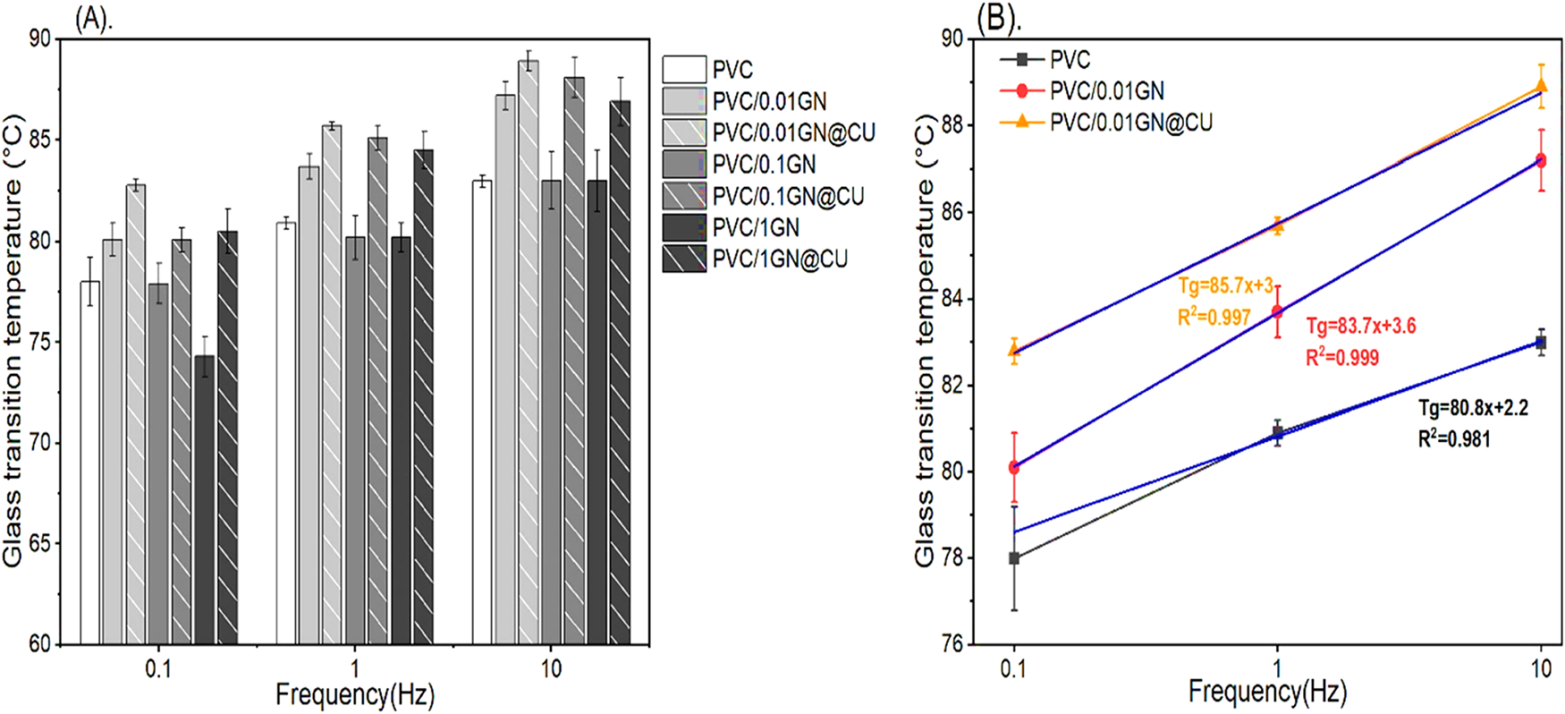
(A) *T*_g_ for pure PVC and PVC nanocomposites containing GN and GN@Cu for frequencies of 0.1, 1, and 10 Hz, (B) linear trend model for materials containing 0.01 wt % fillers.

Figure 5B presents the dependence of the glass transition temperature *T*_g_ on the excitation frequency together with the linear fit. For all investigated materials, a systematic increase in *T*_g_ with increasing frequency is observed, which is a direct consequence of the kinetic nature of the glass transition and the dominance of the α-relaxation process. At higher frequencies, polymer chain segments do not reach full relaxation, and the glass–rubber transition maximum shifts toward higher temperatures.^71,72^ Within the analyzed range, the dependence of *T*_g_ on frequency is well described by a linear model, as confirmed by a high coefficient of determination close to unity. An increase in the slope of the trend line after the introduction of graphene indicates the sensitivity of the glass transition temperature to changing measurement conditions. It can be associated with restricted mobility of PVC chain segments in the vicinity of the filler.^73,74^ The highest slope value was obtained for the nanocomposite containing modified graphene, suggesting more effective matrix–filler interactions and improved graphene dispersion.^75^ Higher intercept values for the nanocomposites compared to neat PVC indicate an increase in the glass transition temperature, related to immobilization of polymer segments in the interfacial region, whereas the slightly lower intercept observed for the modified graphene may reflect partial relaxation of interfacial stresses and a more homogeneous structural character of the material.^76^

The example run of the real modulus *E*′ for frequency 1 Hz and the mechanical loss factor tan δ by DMTA measurements as a function of temperature (A), dependence of changes of dielectric loss factor tan δ_dl_ as a function of temperature (B) as well as DSC thermograms (C) for PVC, PVC/0.01%GN and PVC/0.01%GN@CU nanocomposites are presented in Figure 6, but for nanocomposite samples containing GN and GN@CU with the concentration of 0.1% and 1.0% these dependences are shown in Figures S3–S5 (see Supporting Information).

**Figure 6:**
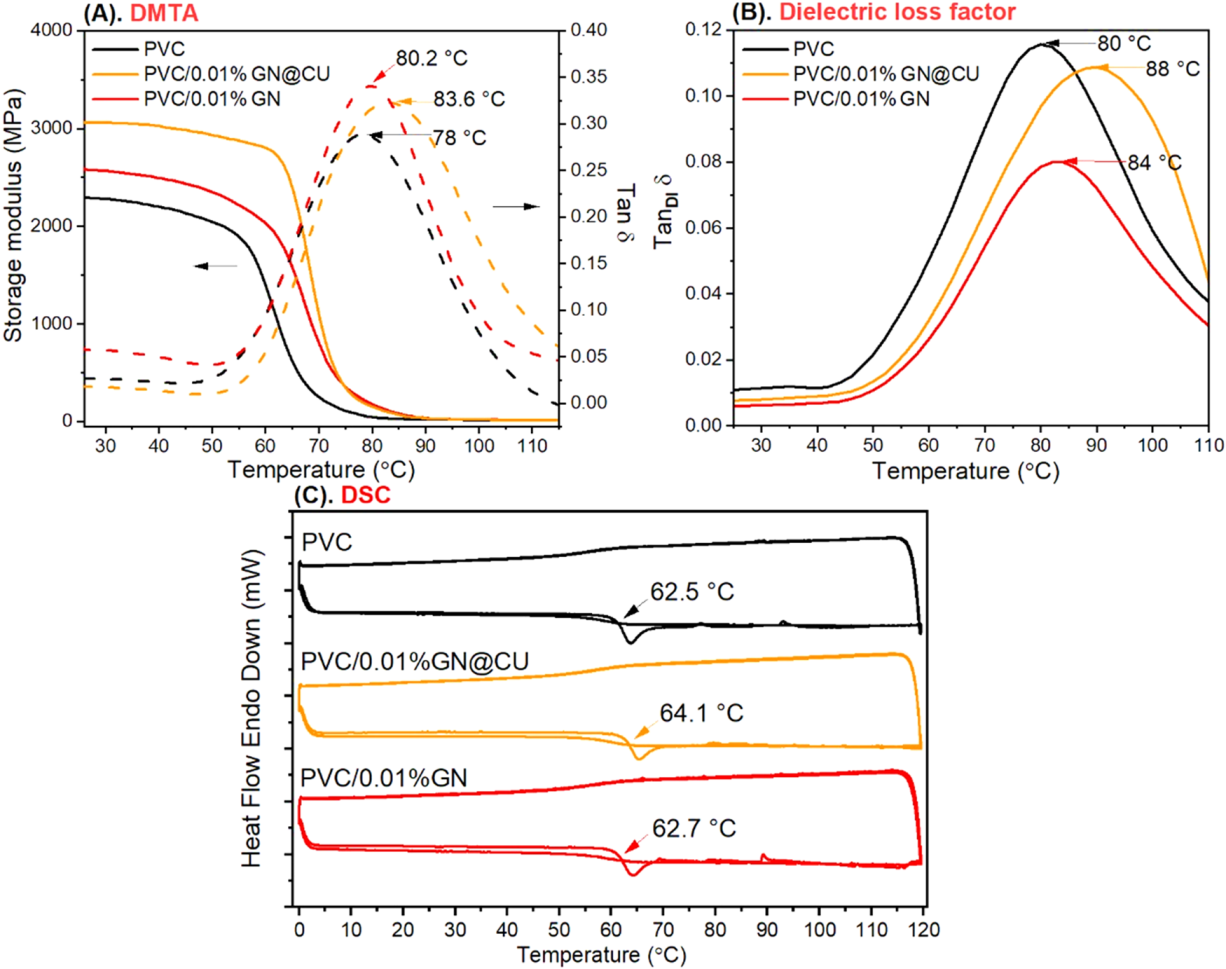
DMTA thermograms, frequency 1 Hz (A) dielectric loss factor vs temperature (B) and DSC thermograms (C) of PVC, PVC/0.01%GN, and PVC/0.01%GN@CU.

From the analysis of the DMTA thermograms shown in Figures 6A and S3, it can be seen that the introduction of both unmodified and surface-modified graphene using curcumin into the PVC matrix results in an increase in the intensity of tan δ, especially in the case of samples with unmodified graphene. At the same time, a shift of the tan δ maximum toward higher temperatures is observed; the *T*_g_ value of PVC/0.01%GN and PVC/0.01%GN@CU nanocomposites is therefore higher compared to PVC by 2.2 and 5.6 °C, respectively. The more than 2-fold increase in glass transition temperature observed for the sample containing 0.01% curcumin-modified graphene is due to the homogeneous dispersion of the nanofiller in the polymer matrix because of its surface modification. The observed effects suggest an interaction between the filler’s surface and the PVC chains, which restricts their mobility. This is confirmed by the temperature-dependent changes in the storage modulus *E*′, which is highest in the temperature range of glass transition for nanocomposites containing graphene modified with curcumin. Thus, improved dispersion homogeneity of graphene within the PVC matrix contributes to increased stiffness of the material. In this case, the decrease in *E*′ value in the glass transition temperature range is more rapid but begins at a higher temperature than PVC and PVC/0.01% GN (see Figure 6A).

The course of the curves of the dependence of dielectric loss as a function of temperature, shown in Figures 6B and S4 of samples of unmodified PVC and nanocomposites containing GN and GN@CU, is similar, independent of the composition. In each case, an increase in the tangent of dielectric loss to a maximum value occurring near the glass transition temperature, associated with an increase in the mobility of the polymer chains with increasing temperature, is observed. With further increase in temperature, the tan δ_DI_ values gradually decrease. The maximum value of the dielectric loss factor tan δ_Dl_ is the highest for the unmodified PVC sample and, at the same time, occurs at the lowest temperature, i.e., about 80 °C, compared to the nanocomposite samples. An increase in the measurement temperature to this value causes an increase in the mobility of polymer chains, which promotes greater energy loss in the electric field. The increase in *T*_g_ value associated with the introduction of 0.01% GN and 0.01% GN@CU into the matrix is 4 and 8 °C, respectively, and indicates the effect of graphene on the mobility of the polymer chains; its reduction is particularly significant in the case of carbon nanofiller with modified surface (Figure 6B).

Additionally, the values of the tangent of dielectric loss of the nanocomposites decrease with the addition of GN and GN@CU, which correlates well with the increase in conductivity of graphene-filled PVC nanocomposites found in our previous work.^30,32^ It should be noted that modification of its surface with curcumin had only a slight effect on the decrease in the tangent of dielectric loss associated with the decrease in chain mobility. This effect is consistent with the trend of changes in surface and volume resistivity described in our work, which decreased significantly only for PVC composites containing more than 0.1% of unmodified GN. No such decrease was found for materials containing graphene modified with turmeric derivatives, despite the observed improvement in structure homogeneity.

Figures 6C and S5 show DSC thermograms. The glass transition appears as a step change or inflection in the baseline heat flow signal, corresponding to a change in the heat capacity of the material. The glass transition temperature *T*_g_ was defined as the temperature at the inflection point (the middle point of this transition region) where the slope of the curve changes most rapidly. The DSC curves of all the samples, regardless of the type and concentration of graphene filler, were similar within the glass transition range. The values of *T*_g_ of unmodified PVC and the nanocomposites with GN and GN@CU ranged from 62.5 °C (PVC) to 64.1 °C (PVC/0.01% GN@CU).

The summarized values of glass transition temperature for PVC and all nanocomposites with GN and GN@CU, determined based on three methods used in our research, i.e., by DMTA, dielectric measurements, and DSC, are shown in Table 1. As can be seen, the PVC matrix always has a lower *T*_g_ value than PVC-based nanocomposites with GN and GN@CU. The differences observed in the glass transition temperature between the composites and the unfilled PVC are more pronounced in dielectric measurements. In these measurements, the temperature difference between the unfilled PVC and the PVC filled with 0.01% GN@CU is approximately 8 °C. Increasing the concentration of unmodified graphene from 0.1% to 1% has virtually no effect on the change in glass transition temperature range as determined from DMTA thermograms; the *T*_g_ values of nanocomposites with GN remain almost identical to those of films containing 0.01%. In the case of nanocomposites with GN@CU, *T*_g_ values are reduced by about 1 °C.

**Table 1:** Glass Transition Values Determined Based on DMTA and DSC Analysis, as well as from Dielectric Loss Factor Measurements

	*T*_g_ (°C) DMTA		*T*_g_ (°C) dielectric loss factor		*T*_g_ (°C) DSC	
filler content (wt %)	GN	GN@CU	GN	GN@CU	GN	GN@CU
0	80.9 (1.2)		80.0 (0.9)		62.5 (1.5)	
0.01	83.7 (0.6)	85.7 (0.2)	84.0 (1.0)	88.0 (0.5)	62.7 (1.8)	64.1 (0.5)
0.1	80.2 (1.1)	85.1 (0.6)	82.0 (1.2)	82.0 (0.7)	61.0 (1.6)	62.4 (0.9)
1	80.2 (0.7)	84.5 (0.9)	82.0 (1.1)	82.0 (0.9)	59.1 (2.1)	61.9 (1.1)

The glass transition temperatures determined from dielectric loss measurements are higher than those determined from DMTA measurements, which confirms the dependence of *T*_g_ on the measurement frequency described in our previous works.^21,22^ At the same time, the glass transition temperatures change slightly differently with increasing nanofiller content; an increase in the concentration of GN and GN@CU to 0.1% and 1.0% results in a decrease in the *T*_g_ value to 82 °C, i.e., by 2 and 6 °C compared to films containing nanofillers at a concentration of 0.01%. Similarly to our studies on the glass transition of PVC modified with oligomeric silsesquixanes and carbon nanotubes, the *T*_g_ values determined by thermal analysis using the DSC method are significantly lower than those determined using DMTA and dielectric loss measurements.^21,22^ The glass transition temperatures of all PVC-based nanocomposites are similar to those of the unmodified polymer, regardless of the concentration and type of filler. Moreover, compared to other methods, the lowest impact of graphene and graphene with modified surface (in terms of the concentrations used) on the *T*_g_ value was observed by the DSC measurements.

In summary, within the range of graphene nanofiller concentrations used, the greatest effect on the glass transition temperature of PVC was observed at 0.01% graphene modified with curcumin. Such an effect was observed regardless of the testing technique used, with the most pronounced response occurring during dielectric testing and the least during thermal analysis. This can be attributed to the material’s different responses to the types of loading at various frequencies used in the measurements. However, the efficiency of graphene surface modification with curcumin is limited, so a decrease in the glass transition temperature was observed in nanocomposite materials containing GN@CU at higher concentrations compared to those containing 0.01% nanofiller, which is related to the greater inhomogeneity of their structure. In any case, however, the glass transition temperature values of materials with curcumin-modified graphene are higher compared to those containing unmodified graphene.

Figures 7 and 8 present the temperature dependence of spin–lattice relaxation time *T*_1_ for pure PVC, as well as for PVC/GN and PVC/GN@CU nanocomposites, respectively. For pure PVC, the relaxation times decrease slightly in the range from 80 to 300 K. At the temperature of 300 K, a discontinuity in relaxation behavior is observed. Above this temperature, the relaxation times decrease rapidly, without reaching a minimum within the high temperature range available in the experiment. It is assumed that the discontinuity in the course of *T*_1_ values is related to the glass transition. In this temperature range, the mobility of the polymer chain segments increases, leading to more efficient relaxation and consequently a shortening of the *T*_1_ time.

**Figure 7:**
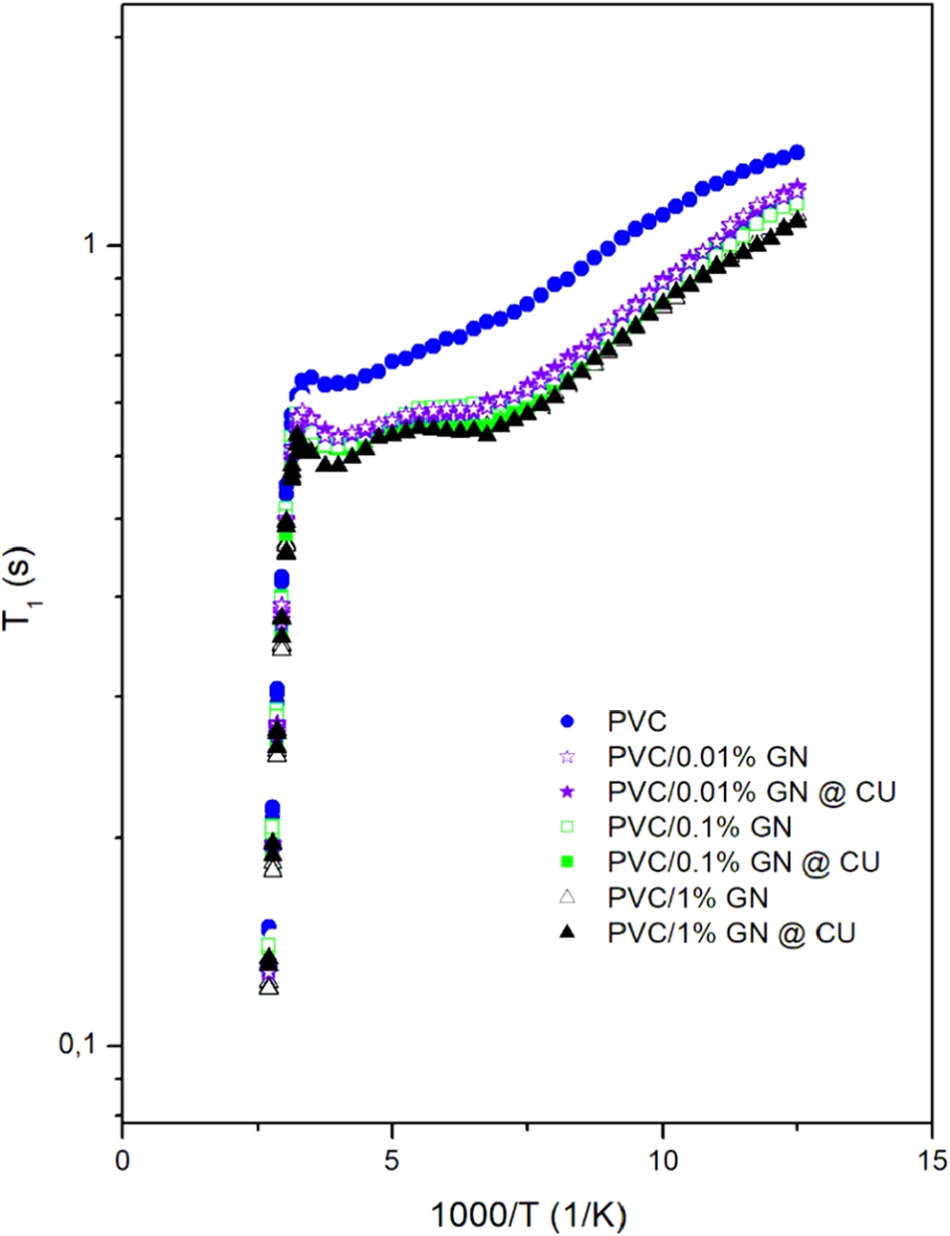
Temperature dependence of the spin-relaxation times *T*_1_ in the laboratory frame for pure PVC, PVC/GN, and PVC/GN@CU nanocomposites.

**Figure 8:**
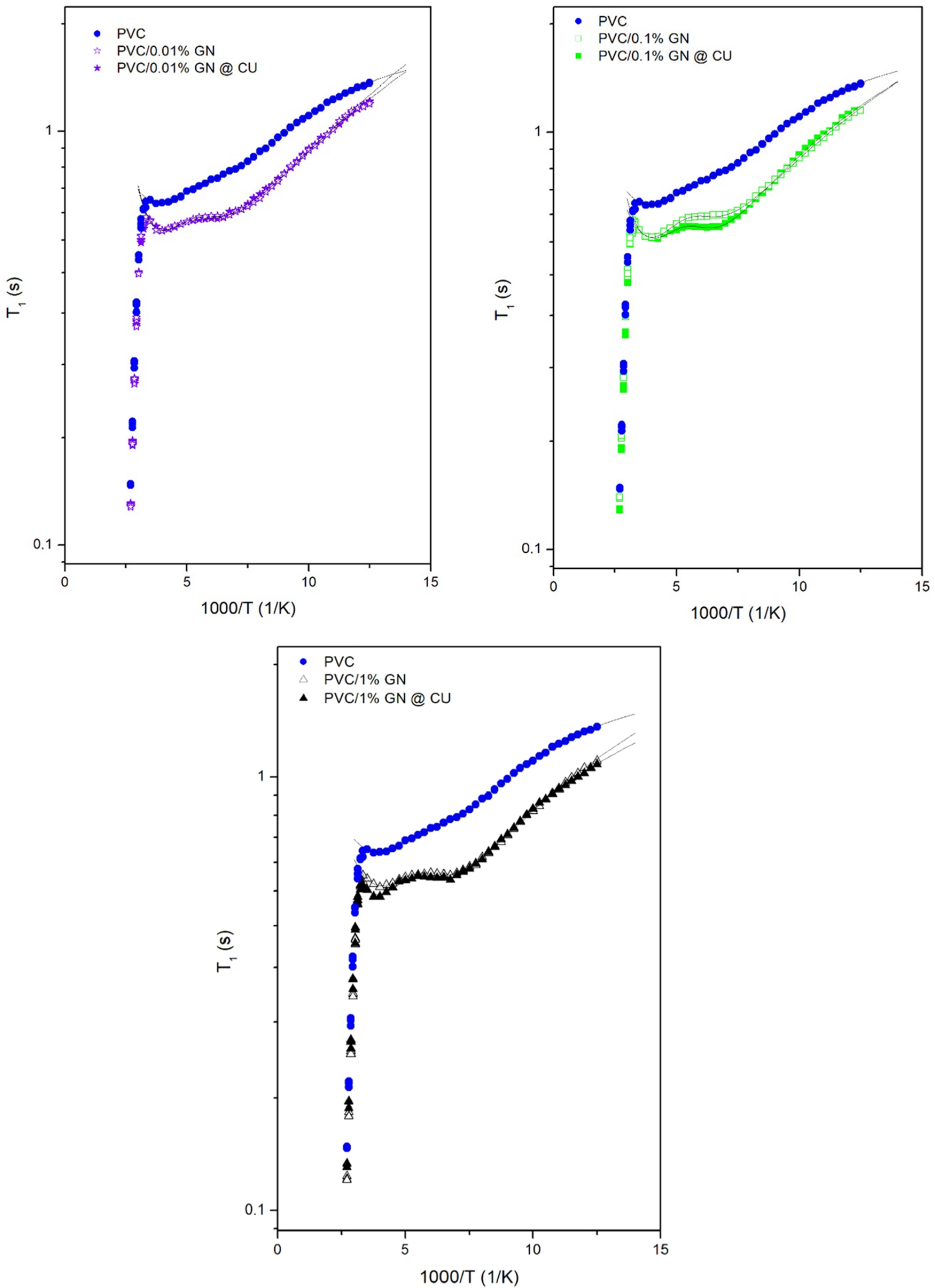
Temperature dependence of the spin-relaxation times *T*_1_ for pure PVC, PVC/GN, and PVC/GN@CU nanocomposites with the best fitting lines.

Analyzing the temperature dependence of relaxation times for nanocomposites, as shown in Figure 8, it was found that the addition of GN and curcumin-modified graphene GN@CU affects the molecular dynamics of PVC, but only in the low temperature range. For all nanocomposites, a shortening of relaxation times compared to pure PVC and the appearance of significantly deeper and shifted toward higher temperatures minima were observed. Above 300 K, the temperature dependence of the relaxation times *T*_1_ is the same for all nanocomposites and pure PVC. Additionally, it was found that the temperature at which discontinuity in the relaxation times occurs shifts slightly toward higher values for all nanocomposites. For PVC/0.01%GN and PVC/0.01%GN@CU nanocomposites, there are no changes in the temperature dependence of the relaxation times *T*_1_. Although the addition of 0.01 wt % of GN or GN@Cu does not result in significant changes in the *T*_1_ relaxation times measured by NMR, distinct effects are observed in DMA, DSC, and dielectric measurements. This discrepancy arises from the different sensitivity and time/frequency scales of these techniques. While NMR probes local molecular motions in the MHz-GHz range and provides an averaged view over the entire sample, techniques like DMA and dielectric spectroscopy are more sensitive to changes in segmental dynamics, interfacial interactions, and localized restrictions in polymer chain mobility. Therefore, even low concentrations of nanofillers may induce detectable changes in thermal and mechanical behavior, without significantly affecting spin–lattice relaxation.

Figure 7 shows that, for PVC sample containing 0.1% GN@CU, the *T*_1_ relaxation times in the temperature range from 125 to 210 K are shorter than those for PVC containing 0.1% GN. The low-temperature minimum for GN@CU sample is deeper, narrower, and shifted toward higher temperatures. Analyzing the temperature dependence of the *T*_1_ relaxation times for PVC nanocomposites with a 1% filler content, a noticeable difference is observed only in the temperature range from 208 to 303 K, where the sample containing the GN@Cu filler exhibits a deeper minimum. The shift in this temperature of about 2–3 K for the nanocomposite suggests restricted mobility of PVC chain due to interfacial interactions between the PVC matrix and graphene GN nanofiller.^10,11,77,78^

The molecular dynamics of poly­(vinyl chloride) at temperatures significantly below the glass transition temperature *T*_g_ is dominated by restricted, localized motions of polymer chain segments. Using solid-state NMR and broadband dielectric spectroscopy (BDS), the relaxation processes below *T*_g_ have been identified, corresponding to rapid, low-energy vibrations of functional groups such as −CH_2_– and −CHCl–, with activation energies ranging from 5 to 25 kJ/mol. Additionally, torsional motions and restricted rotations around single C–C bonds, particularly near chain ends, with activation energies in the range of 10 to 40 kJ mol^–1^, were also observed.^79^

At temperatures lower than the glass transition temperature *T*_g_, segmental α-relaxation and translational mobility are suppressed. Analysis of spin–lattice relaxation times *T*_1_ confirms the presence of thermally activated, localized molecular motions despite the rigid amorphous matrix of PVC. The minimum associated with the glass–rubber transition is not accessible in the temperature range available for the NMR technique. It is expected that only local motion of the main chain is detected in the temperature range between 80 and 300 K, above which a sharp decrease in the *T*_1_ relaxation times occurs. It is assumed that in the low-temperature region below the *T*_g_ temperature for PVC there are two shallow minima related to local motions, the depth of which increases with the addition of graphene or graphene modified with curcumin.

Activation parameters describing the molecular dynamics of PVC and its nanocomposites can be determined by analyzing the temperature dependence of the spin-relaxation times *T*_1_, based on the dipole–dipole Bloembergen-Purcell-Pound (BPP) theory.^80^

It is assumed that below glass transition temperature, *T*_1_ values are influenced by dipolar interactions modulated by local motions of both molecular groups and polymer chain segments. Since these contributions are additive, *T*_1_ can be determined using the following formula^81,82^

1 $$\frac{1}{T_{1}}=\frac{2}{3}\gamma^{2}\sum_{i}\Delta M_{2i}\left[J_{i}\left(\omega \right)+4J_{i}\left(2\omega \right)\right]$$

where: γ represents the proton gyromagnetic ratio,, Δ*M*_2*i*_ corresponds to the decrease in the second moment observed in the ^1^H NMR spectra due to molecular motions in the system, and *J_i_* (ω) refers to the spectral density function that characterizes the frequency-dependent contribution of molecular motions to the relaxation mechanism.

In polymeric systems, the minima of the relaxation times *T*_1_ are often very broad and asymmetric, reflecting a wide and complex distribution of molecular motions. To accurately characterize these relaxation processes, it is necessary to introduce a distribution of correlation times that accounts for the system’s heterogeneity. The spectral density function proposed by Davidson and Cole was employed to determine the spectral density function *J*(ω) in eq 2 ^83^

2 $$J\left(\omega ,\tau_{c},\beta \right)=\frac{2}{\omega }\left[\frac{\sin\left(\beta \mathrm{arc}\tan\left(\omega \tau_{c}\right)\right)}{{\left(1+\omega^{2}\tau_{c}^{2}\right)}^{\beta /2}}\right]$$

where: τ_c_ is the upper cutoff characteristic correlation time, while the parameter β (0 < β< 1) describes the width and asymmetry of the correlation time distribution.

Assuming that the observed motions follow thermally activated behavior, the correlation time τ_c_ varies with temperature according to the Arrhenius equation

3 $$\tau_{c}=\tau_{0}\exp\left(-\frac{E_{a}}{\mathrm{RT}}\right)$$

In eq 3, τ_0_ represents the pre-exponential factor, *E*_a_ denotes the activation energy, and *R* refers to the universal gas constant.

The theoretical curves presented in Figure 8 correspond to the best fits to eq 1, based on the relationships defined in eqs 2 and 3, applied to the experimental data. The corresponding fitting parameters are summarized in Tables 2 and 3. Activation parameters, including the pre-exponential factor τ_0_, activation energy *E*_a_, and the distribution width of correlation times β, describing two distinct local molecular motions were determined for pure PVC, as well as PVC/GN, and PVC/GN@CU nanocomposites.

**Table 2:** Motional Parameters Obtained for Pure PVC and PVC/GN Nanocomposites by Fitting Theoretical Relaxation Curves to Experimental Data^a^

	the first local motion in the low temperature				the second local motion in the low temperature			
material	τ_0_ × 10^–12^ (s)	*E*_a_ (kJ/mol)	*B*	*M*_2_ (10^–8^T^2^)	τ_0_ × 10^–10^ (s)	*E*_a_ (kJ/mol)	B	*M*_2_ (10^–8^T^2^)
PVC	11.2	8.1	0.1	4.49	6.9	4.6	0.7	0.4
PVC/0.01%GN	8.0	8.7	0.1	1.01	0.7	12.0	0.2	1.0
PVC/0.1%GN	2.7	9.9	0.1	1.53	1.0	10.9	0.2	1.0
PVC/1%GN	4.5	9.2	0.1	1.48	4.0	6.1	0.5	0.5

a Uncertainties of the estimated parameters are lower than 10%.

**Table 3:** Motional Parameters Obtained for Pure PVC and PVC/GN@CU Nanocomposites from Fitting the Theoretical Relaxation Curves to Experimental Data

	the first local motion in the low temperature				the second local motion in the low temperature			
material	τ_0_ × 10^–12^ (s)	*E*_a_ (kJ mol^–1^)	*B*	*M*_2_ (10^–8^T^2^)	τ_0_ × 10^–10^ (s)	*E*_a_ (kJ mol^–1^)	B	M_2_ (10^–8^T^2^)
PVC	11.2	8.1	0.1	4.5	6.7	4.6	0.7	0.4
PVC/0.01%GN@CU	17.9	7.9	0.1	1.1	1.3	9.6	0.3	0.7
PVC/0.1%GN@CU	2.6	10.2	0.1	1.6	3.4	6.4	0.5	0.5
PVC/1%GN@CU	1.3	11.1	0.1	2.2	3.7	6.5	0.5	0.6

The incorporation of graphene GN into poly­(vinyl chloride) PVC matrix leads to a notable reduction in spin–lattice relaxation times *T*_1_, as shown in Figures 7 and 8. This decrease in *T*_1_ indicates more efficient energy transfer between nuclear spins and the lattice, primarily due to the restricted mobility of polymer chains in the vicinity of the graphene surface. Strong interfacial interactions between the π-conjugated surface of graphene and PVC, along with possible magnetic field inhomogeneities introduced by the graphene layers, enhance local dipolar interactions, thereby accelerating the relaxation process. Additionally, if the graphene contains residual metal catalysts or defects, it may introduce paramagnetic centers, which further contribute to a significant reduction in relaxation times. As a result, the shortened *T*_1_ values reflect a more rigid and constrained polymer environment, which correlates with the observed improvements in thermal and mechanical performance of PVC/GN nanocomposites. The improved dispersion and interfacial bonding in PVC/GN systems leads to reduced chain mobility and faster relaxation, as confirmed by the higher values of activation energy and deeper minima of *T*_1_ relaxation times for PVC/GN nanocomposites in comparison to pure PVC.^28,34,38,84^

The incorporation of graphene and curcumin into the PVC matrix also significantly affects the mobility of PVC chains, primarily due to improved dispersion and enhanced interfacial interactions. The curcumin-functionalized graphene GN@CU demonstrates better dispersion within the PVC matrix as a result of strong π–π interactions and hydrogen bonding, which also contributed to improved interfacial adhesion. As a consequence, the nanocomposites exhibited an increase in the glass transition temperature by several degrees, indicating reduced mobility of the polymer chains.^34^ he activation parameters obtained for pure PVC and PVC/GN@CU nanocomposites are presented in Table 3. Higher activation energy values for PVC/GN@CU nanocomposites in comparison to pure PVC and PVC/GN nanocomposites and shorter the pre-exponential relaxation time τ_0_ can be interpreted that the addition of nanofiller restricted the motion of polymer chain segments on a local scale.

Relaxation behavior in PVC/GN and PVC/GN@CU nanocomposites reflects the altered mobility of polymer chain segments due to the presence of graphene or curcumin-modified graphene as a nanofillers. The observed shift of the relaxation minima toward higher temperatures and changes in activation parameters indicate restricted molecular motions caused by interfacial interactions and physical immobilization of PVC segments near the graphene surface. The addition of graphene results in a decrease in the pre-exponential relaxation time τ_0_, an increase in activation energy *E*_a_, and a broadening of the correlation time distribution, which together point to enhanced dynamic heterogeneity within the system. These changes confirm that graphene acts as a stiffening agent, affecting local motions of PVC chains.

Glass transition studies demonstrated that the incorporation of graphene into the PVC matrix leads to restricted mobility of polymer chain segments, manifested by an increase in the *T*_g_ value compared to neat PVC, with this effect being particularly pronounced for nanocomposites containing curcumin-modified graphene. The observed changes in *T*_g_ indicate strengthened interactions in the polymer–filler interfacial region, resulting in partial immobilization of PVC macromolecules. XPS analysis presented in Figure S6 and Tables S2 and S3 (see Supporting Information) confirms that these interactions are of a physical nature, as the absence of significant shifts in the C–Cl binding energy and the lack of new chemical components exclude the formation of covalent bonds between PVC and graphene. At the same time, nanocomposites containing GN@CU exhibit changes in the relative contributions of C–O and C=O groups and an oxygen content, indicating modification of interface area of the two phases and promoting improved adhesion and dispersion of the filler within the PVC matrix. Consequently, the strengthening of polymer–filler interactions should be attributed not to their chemical character, but to an increased effective interfacial contact area and intensified physical interactions, which is directly reflected in reduced segmental dynamics of PVC and an elevated glass transition temperature of the nanocomposites.

## 4 Conclusions

The glass transition of poly­(vinyl chloride) is an issue not fully recognized, especially in terms of the influence of various types of modifiers used to improve its functional properties, such as electrical conductivity through the addition of carbon nanofillers. Understanding the mechanisms of interactions between polymer macromolecules and nanoparticles at the molecular level is crucial for designing materials tailored to specific applications.

For this reason, supplementing conventional methods for determining the glass transition temperature with NMR techniques, which allow the assessment of chain local and segment mobility, is particularly valuable for characterizing these interactions on a molecular scale. In this study, noncovalent modification of the graphene surface with curcumin, occurring via a π-π mechanism, enhanced these interactions. It results from increased contact area at the filler–polymer interface due to improved filler dispersion in the matrix. The consequence of enhanced interactions is an increase in both the glass transition temperature and the stiffness of the composite material.

Enhancing polymer–filler interactions is important in this context in terms of the application of new engineering materials for the production of structural components that can be heated under service conditions, and it is required to maintain dimensional stability and mechanical properties, especially stiffness.

Dynamic techniques that probe macromolecular mobility and their response to mechanical loading and varying electric fields are, therefore, especially valuable for assessing temperature-dependent changes related to the glass transition in PVC-based nanocomposites containing graphene fillers.

## Supplementary Material



## Data Availability

The data that support the findings of this study are available from the corresponding author upon reasonable request.

## Notes

Materials include XPS spectra for graphene, curcumin-modified graphene (Figures S1 and S2), and PVC-based nanocomposites containing 1 wt % of both types of nanofillers (Figure S6); the bond energies and atomic percentages for GN and GN@CU determined from the spectra are summarized in Table S1, and for the PVC/1%GN, PVC/1%GN@CU, and PVC nanocomposites in Tables S2 and S3; DMTA thermograms for nanocomposites containing 0.1 and 1 wt % graphene and curcumin-modified graphene, respectively, shown in Figure S3; Figure S4 contains the results of dielectric loss angle as a function of temperature for these materials, and the results of thermal analysis by DSC are presented in Figure S5 (PDF)
