# Supplementary material for: Graphene Modified with Curcumin: A Novel Approach to Tailoring the Glass Transition of PVC
Source: J Phys Chem B. 2026 Feb 19;130(9):2637–51. doi: 10.1021/acs.jpcb.5c06706 (PMC13362196; doi:10.1021/acs.jpcb.5c06706)
Supplement: Supplementary file 1 [file jp5c06706_si_001.pdf]

Graphene Modified with Curcumin: A Novel Approach to Tailoring the Glass Transition of PVC

Sławomir Wilczewski[1], Aneta Woźniak-Braszak*[2], Paweł Bilski[2], Jolanta Tomaszewska*[1]

[1]Bydgoszcz University of Science and Technology, Faculty of Chemical Technology and Engineering, Seminaryjna 3, 85-326, Bydgoszcz, Poland
[2]Faculty of Physics and Astronomy, Adam Mickiewicz University, Uniwersytetu Poznańskiego 2, 61-614 Poznań, Poland

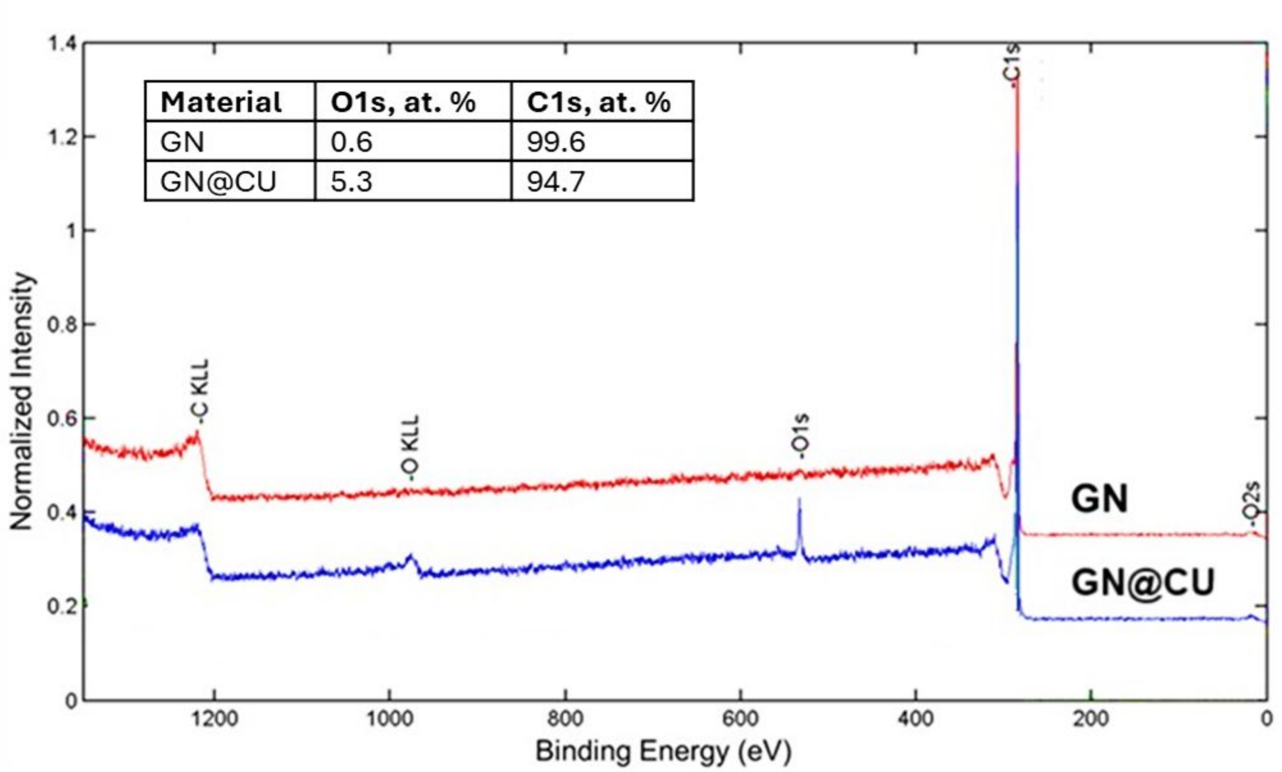

Fig. S1. XPS spectra of the GN and GN@CU.

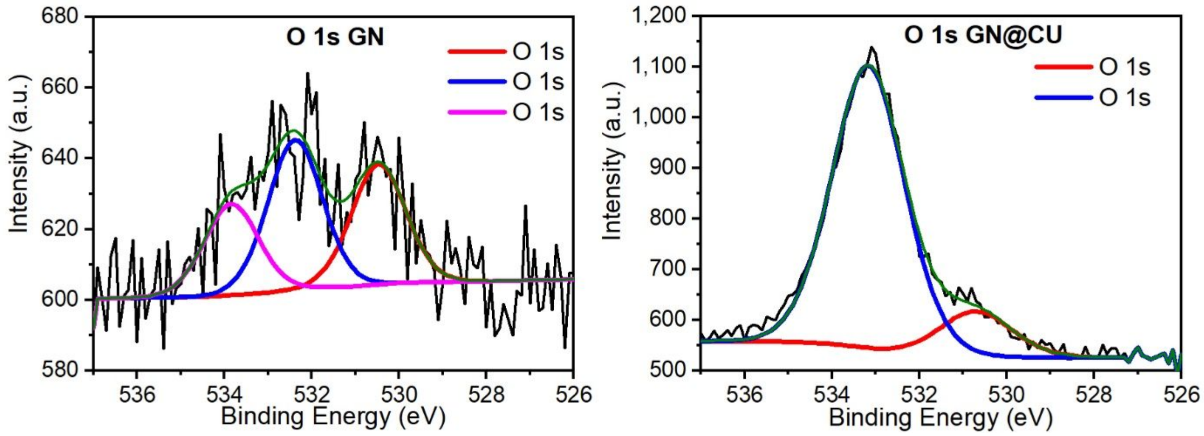

Fig. S2. O1s peaks deconvolution.

Table S1. XPS characterization of GN and GN@CU

Binding energy, eV	Chemical Bonds	FWHM	Atomic, %
	**GN**		
530.47	O1s	1.47	0.2
532.38	O1s	1.47	0.2
533.84	O1s	1.47	0.2
			**0.6**
284.53	C sp^2	0.82	65.3
285.33	C sp^3	0.82	7.5
286.11	C-O	1.49	10.0
287.56	C=O	1.49	4.5
289.21	COOR	1.49	3.5
290.68	π electrons	1.49	4.6
292.03	sp^2 loss shakeup	1.49	2.8
293.87	sp^2 loss shakeup	1.49	1.1
			**99.4**
	**GN@CU**		
530.7	O1s	1.95	0.7
533.17	O1s	1.95	4.6
			**5.3**
284.42	C sp^2	0.73	50.9
284.81	C sp^3	0.73	9.6
285.72	C-O	1.82	16.5
287.18	C=O	1.82	6.4
288.99	COOR	1.82	4.0
290.73	π electrons	1.82	4.2
292.14	sp^2 loss shakeup	1.82	2.1
294.15	sp^2 loss shakeup	1.82	1.0
			**94.7**

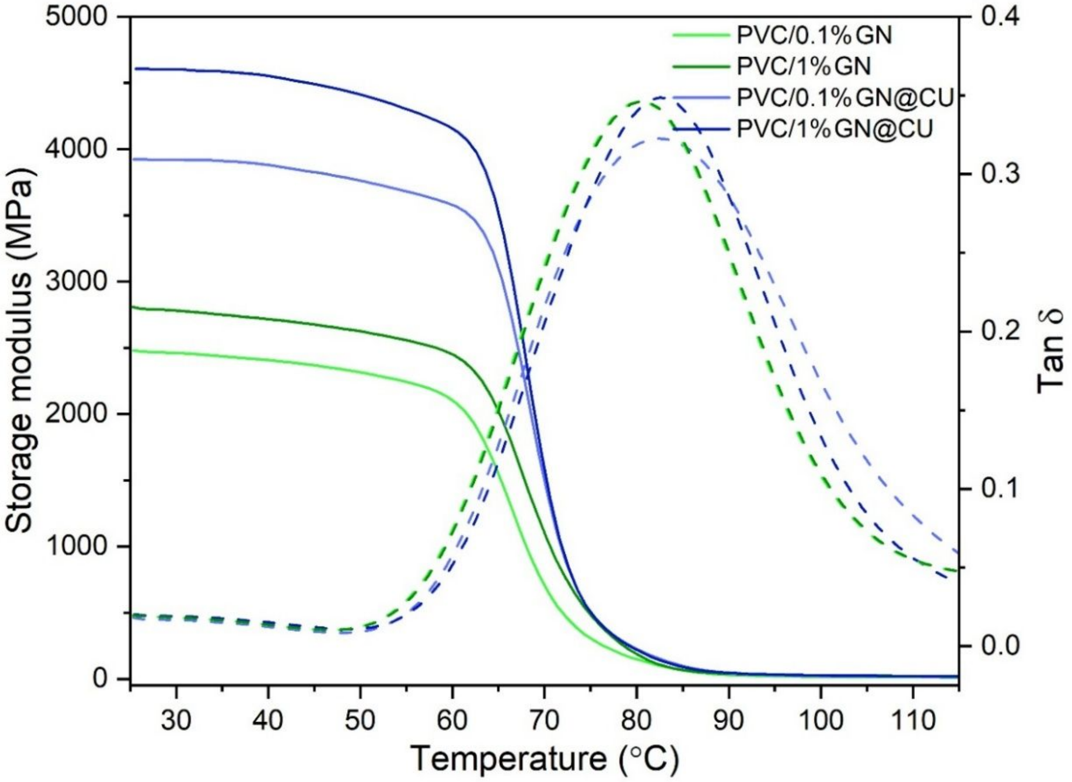

Fig. S3. DMTA analysis.

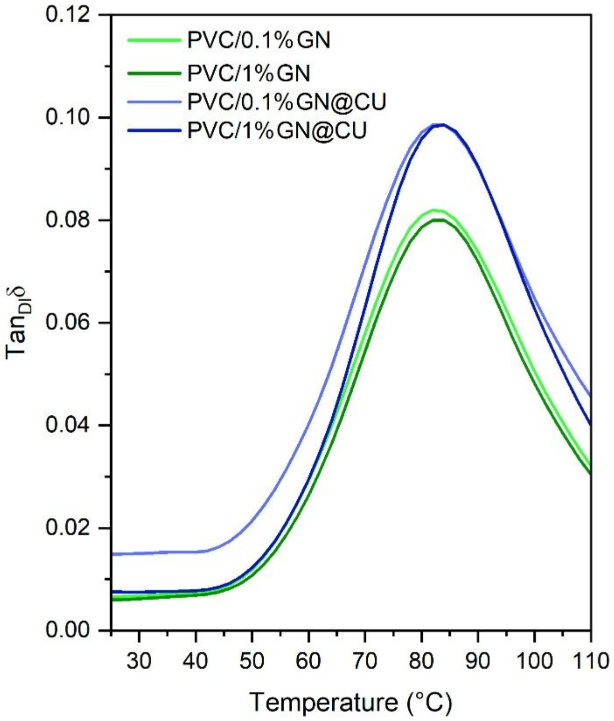

Fig. S4. Dielectric loss factor vs. temperature.

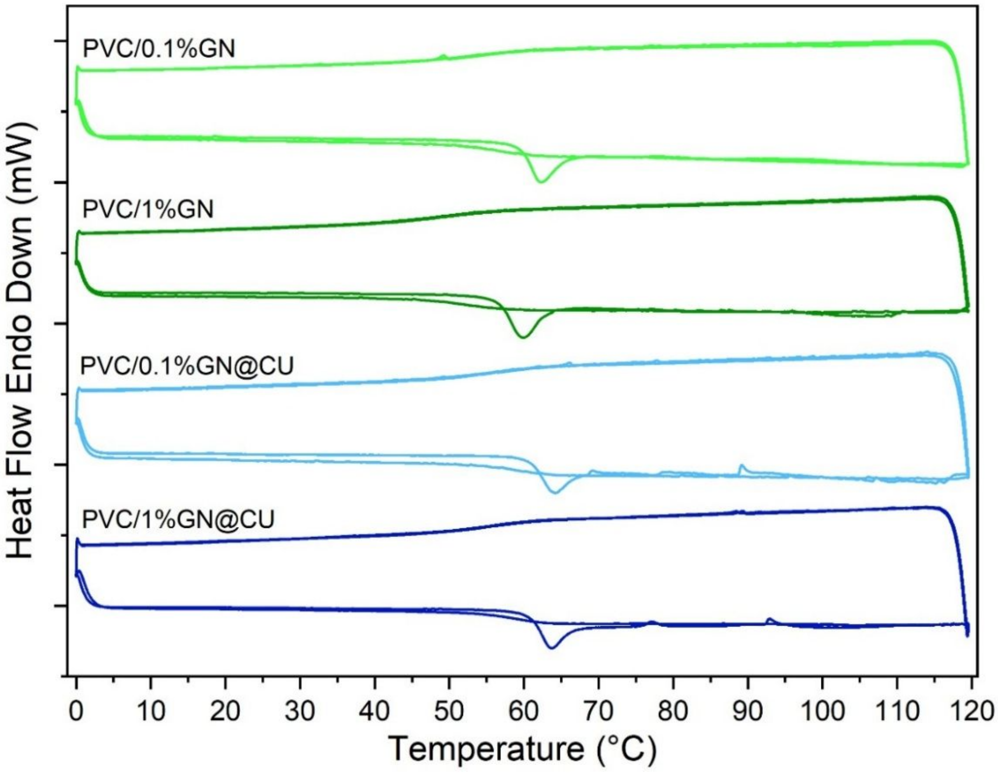

Fig. S5. DSC analysis.

Table S2. Atomic % of bonds in PVC, PVC/1%GN and PVC/1%GN@CU

	O=C	O-C	C-C	C-Cl	C-O	C=O	C1	Cl	Si
PVC	7,25	1,58	36,99	18,08	3,43	1,78	10,21	16,59	4,09
PVC_1GN	5,14	1,61	34,11	20,82	3,72	1,42	11,20	19,39	2,59
PVC_1GN-Cu	5,20	1,54	34,83	18,99	4,35	1,13	11,50	19,97	2,49

Table S3. Atomic % of elements in PVC, PVC/1%GN and PVC/1%GN@CU

	%O	%C	%Cl	%Si
PVC	8,83	70,49	16,59	4,09
PVC_1GN	6,75	71,27	19,39	2,59
PVC_1GN-Cu	6,74	70,80	19,97	2,49

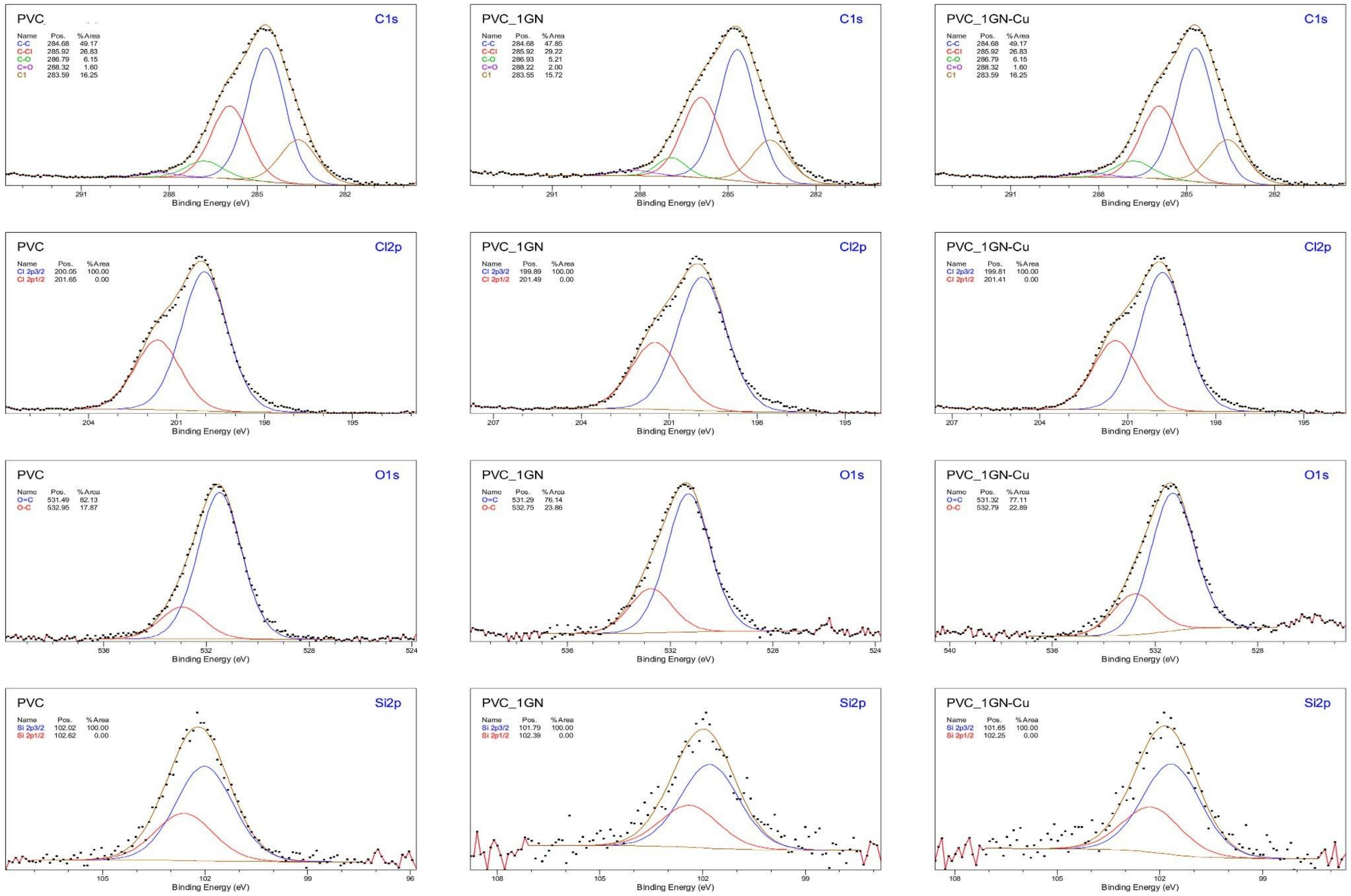

Fig. S6. XPS spectra of the PVC, PVC/1%GN and PVC/1%GN@CU
